# Comparison of a new swept-source optical biometer with a partial coherence interferometry

**DOI:** 10.1186/s12886-018-0936-6

**Published:** 2018-10-19

**Authors:** Hyo Kyung Lee, Mee Kum Kim

**Affiliations:** 1 Department of Ophthalmology, Seoul National University College of Medicine, 103 Daehak-Ro, Jongno-Gu, Seoul, 110-799 Republic of Korea; 2 Laboratory of Ocular Regenerative Medicine and Immunology, Seoul Artificial Eye Center, Seoul National University Hospital Clinical Research Institute, Seoul, Republic of Korea

**Keywords:** Swept-source optical coherence tomography, IOLMaster 700, Optical biometer, Intraocular lens power calculation

## Abstract

**Background:**

The purpose of this study is to compare the biometric parameters and intraocular lens (IOL) power calculation by a new swept-source optical coherence tomography (SS-OCT) biometer with those by a partial coherence interferometry (PCI) biometer.

**Methods:**

Medical records of 175 eyes from 175 patients were retrospectively reviewed. One of two monofocal IOLs (Tecnis ZCB00 or Acrysof SA60AT) were implanted in the eyes. Axial length (AL), mean keratometry (Km), J0, J45 and anterior chamber depth (ACD) were compared between PCI and SS-OCT biometers. The refractive mean error (ME) and refractive mean absolute error (MAE) were also compared. Examination failure rates were calculated in each device.

**Results:**

Out of 175 eyes, 150 eyes were successfully examined by both devices. AL was measured slightly shorter when using SS-OCT than PCI biometer, while Km was measured higher (***P*** < .0001, ***P*** = .03, respectively, paired *t*-test). J0, J45 and ACD were not significantly different between two devices. ME and MAE calculated using SRK-T, Hoffer Q, and Haigis formula were not significantly different except MAE calculated with Haigis formula for Tecnis ZCB00 IOLs (***P*** = .03, paired *t*-test). The examination failure rates were 14.29 and 1.14% when using the PCI and SS-OCT biometers, respectively.

**Conclusions:**

AL and Km don’t seem to be comparable between two devices, while J0, J45_,_ and ACD do. IOL power calculation using SRK-T and Hoffer Q was correlated between the devices. The penetration ability of a SS-OCT biometer is superior.

## Background

As technology has been developed to produce better refractory outcomes, cataract surgery has become a part of refractory surgery [1]. For an accurate intraocular lens (IOL) power calculation, accurate ocular biometry, use of an appropriate calculation formula, and careful optimization of the individual component parts should be considered. Of those, the most important factor is the accuracy of ocular biometric measurements [2]. In the current market, various types of biometric devices based on different technologies are available to provide more accurate ocular biometric measurements.

To our knowledge, a partial coherence interferometry (PCI)-based optical biometer is considered as gold standard for axial length (AL) measurement [3]. In a PCI biometer, optical length is measured from the anterior surface of the cornea to the retinal pigment epithelium with a 780 nm laser diode infrared light. Keratometry (K) is calculated from measurements taken at 6 reference points on the corneal surface in an optical zone with a diameter of 2.4 mm. Anterior chamber depth (ACD) is measured with the lateral slit illumination technique. However, in some cases such as subcapsular lens opacity, dense cataracts, and poor fixation, measuring AL is impossible. For these reasons, the examination failure rate when using a PCI biometer has been reported to be as high as 35.47% [4].

Recently, a swept-source optical coherence tomography (SS-OCT)-based optical biometer was introduced to the market. The SS-OCT technology enables a 44 mm scan depth with 22 μm resolution in corneal tissue using a rapid-cycle, tunable wavelength laser source. Compared with a PCI biometer, an SS-OCT biometer can scan a deeper area and produce a better quality image [5]. Also, it allows for cross-sectional visualization along the visual axis and shows good cataract penetration [6].

The present study was performed to compare biometric parameters and IOL power calculations by a new SS-OCT biometer, the IOLMaster 700® (Carl Zeiss Meditec AG), with those by a PCI biometer, the IOLMaster 500® (Carl Zeiss Meditec AG). Penetration ability was also compared between the two devices.

## Methods

### Patients and methods

The protocol of this study adhered to the tenets of the Declaration of Helsinki. This study protocol was approved and the need for informed consent was waived by our Institutional Review and Ethics Board (No. 1684–174-790). The medical records of the patients who underwent uneventful cataract surgery at Seoul National University Hospital, Seoul, Korea from June 2016 to January 2017 were retrospectively reviewed. Subjects who had been followed for more than 1 month after the surgery were included. The following cases were excluded: those with eventful surgeries, postoperative complications, and other accompanying ocular pathologies such as zonular weakness or macular lesions.

Before the surgery, all individuals were examined in detail using both optical biometers in a random order. The optical parameters of AL, mean K (Km), J0, J45 and ACD were recorded and compared between the devices. Km was calculated as the average of flat K and steep K. Astigmatism was evaluated using Jackson cross-cylinder such as J0 and J45, calculated by power vector analysis [7]. The formula to calculate Jackson cross-cylinder was as follows;

$$ \mathrm{J}0=-\mathrm{c}/2\times \cos 2\theta . $$

$$ {\mathrm{J}}_{45}=-c/2\times \sin 2\theta $$

(c: negative astigmatism = flat K – steep K; *θ* = flat meridian)

To determine the appropriate IOL power, four formulas, SRK-T, Hoffer Q, Holladay, and Haigis, were employed, and the results were compared. Among the formulas, the embedded Holladay formulas were different between the devices used in the present study. Whereas the Holladay 1 formula was embedded in the PCI biometer, the Holladay 2 formula was embedded in the SS-OCT biometer.

A single experienced surgeon (MKK) performed cataract surgeries on all participants enrolled in the present study with a 2.7 mm long, steep-on axis incision technique. For all eyes, one of the two types of monofocal IOLs (Tecnis ZCB00, Acrysof SA60AT) was inserted into the bag without any complications. Target refractive error was from emmetropia to − 2.0 Dsph. We determined the power of the implanted IOL whose predicted refractive error is the targeted one or the negative refraction which is the closest to the emmetropia. The differences between the predicted errors were compared as the refractive mean error (ME) and refractive mean absolute error (MAE), respectively, at 1 week and 1 month after the surgery. ME was defined as the difference between postoperative and predicted spherical equivalents.

### Statistical analysis

The paired *t*-test was used to compare preoperative biometric parameters between the two devices. Agreement of the parameters was evaluated with a Bland-Altman plot. To assess the accuracy of IOL power calculation, ME and MAE were compared between the devices with paired *t*-test 1 week and 1 month postoperatively. The examination failure rate was calculated for each device to compare penetration capability. All statistical tests were performed using SPSS version 18.0 (SPSS Inc., Chicago, Illinois, USA). Statistical significance was defined as ***P*** < .05.

## Results

A total of 175 eyes from 175 patients were enrolled in this study. Twenty-five eyes were excluded from the main analysis because examination by at least one device had failed. Finally, 150 eyes from 150 patients were eligible for the main analysis. The study subjects consisted of 54 men and 96 women. Their mean age was 69.49 ± 9.55 years. Tecnis ZCB00 and Acrysof SA60AT IOLs were implanted in 102 and 48 eyes, respectively.

**Fig. 1:**
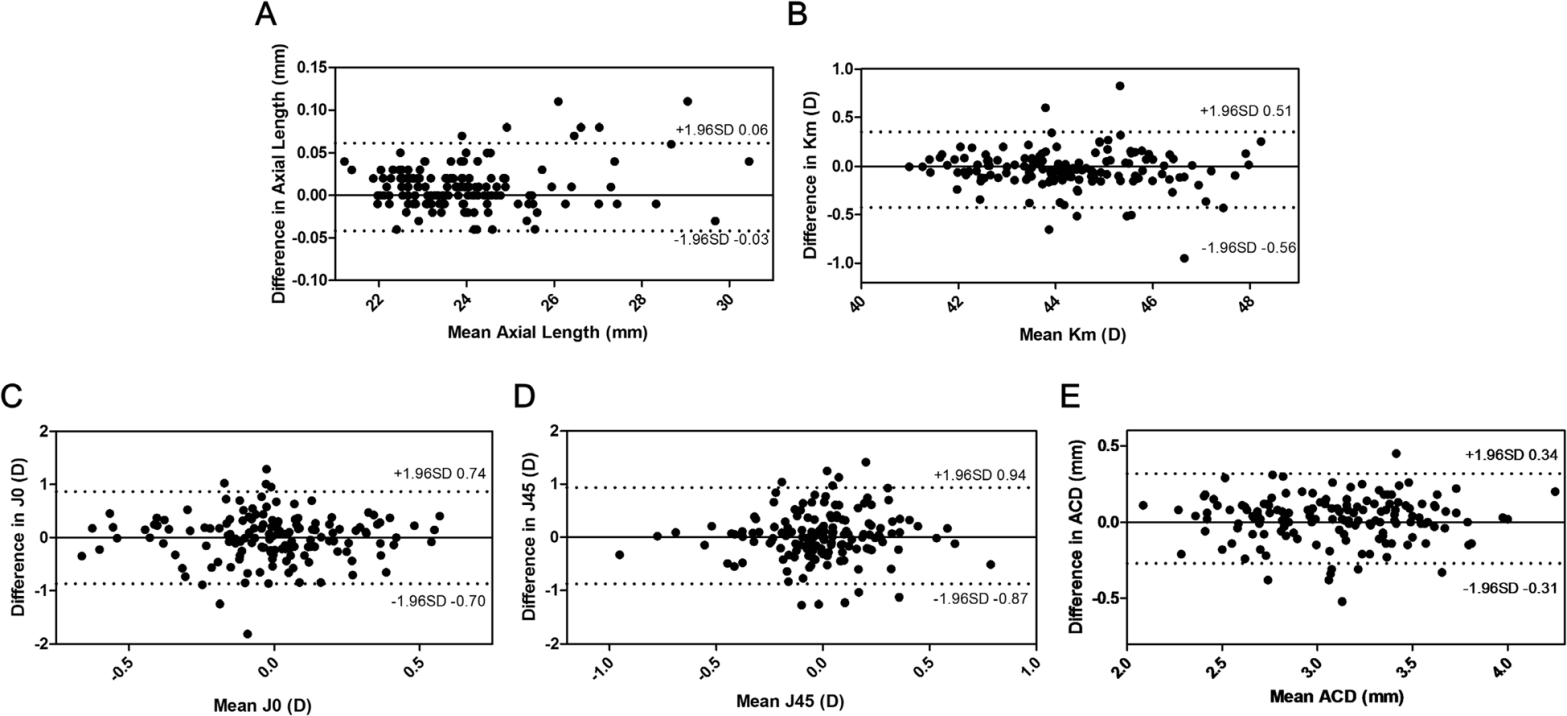
Bland-Altman plots for optical parameters. Bland-Altman plots of the differences in AL (**a**), Km (**b**), J0 (**c**), J45 (**d**) and ACD (**e**) between the SS-OCT and PCI biometers. The middle line indicates the mean difference. The top and bottom dashed lines show the upper and lower 95% LoAs, respectively (AL = axial length; Km = mean keratometry; ACD = anterior chamber depth; SS-OCT = swept-source optical coherence tomography; PCI = partial coherence interferometry; LoA = limit of agreement)

Table 1 shows the ocular parameters obtained by the two devices. The AL were measured as 23.99 ± 1.61 mm and 23.98 ± 1.60 mm when using a PCI and SS-OCT biometers, respectively. The AL measured by a SS-OCT was significantly shorter than a PCI biometer, with a mean difference of 0.0098 ± 0.03 mm (***P*** < .0001, paired *t*-test). Km was measured significantly higher by SS-OCT with a mean difference of − 0.0365 ± 0.20 D (***P*** = .03, paired *t*-test). The two devices provided comparable values for J0, J45 and ACD without significant differences (***P*** = .96, .41, and .06, respectively, paired *t*-test). Figure 1 shows Bland-Altman plots illustrating good agreement for all parameters.

**Table 1:** Comparison of biometric measurements between two devices (*n* = 150)

Parameter	PCI biometer		SS-OCT biometer			
	Mean ± SD	Range	Mean ± SD	Range	Difference	*P* value^*^
AL (mm)	23.99 ± 1.61	21.23 30.47	23.98 ± 1.60	21.19 30.43	0.0098 ± 0.03	< .0001
Km (D)	44.20 ± 1.53	40.99 48.36	44.24 ± 1.55	40.99 48.10	−0.0365 ± 0.20	.03
J0 (D)	−0.007 ± 0.33	−0.995 0.772	− 0.005 ± 0.32	−0.794 0.811	− 0.0019 ± 0.44	.96
J45 (D)	0.008 ± 0.35	−1.117 0.908	−0.022 ± 0.33	−0.789 1.041	0.0309 ± 0.46	.41
ACD (mm)	3.11 ± 0.41	2.14 4.35	3.08 ± 0.41	2.03 4.15	0.0232 ± 0.15	.06

*PCI* partial coherence interferometry, *SS-OCT* swept-source optical coherence tomography, *SD* standard deviation, *AL* axial length, *Km* mean keratometry, *ACD* anterior chamber depth

^*^Paired *t*-test

To assess the accuracy of IOL power calculation, ME and MAE at 1 week (data not shown) and 1 month postoperatively were compared between the two devices (Tables 2 and 3). For Tecnis ZCB00 IOLs, ME was not significantly different between the devices in the SRK-T, Hoffer Q, and Haigis formulas. However, ME in Holladay formula showed a significant difference at 1 week and 1 month postoperatively (all ***P*** < .0001, paired *t*-test). MAE for Tecnis ZCB00 IOLs was not significantly different between two devices in all the formula except Haigis which was significantly lower in SS-OCT (***P*** = .03, paired *t*-test). Meanwhile, for Acrysof SA60AT IOLs, ME and MAE in all the formula at postoperative 1 week and 1 month were not significantly different between the devices except MAE in Holladay formula (***P*** = .02, paired *t*-test).

**Table 2:** Comparison of refractive mean error^a^ at 1 month after the surgery

Formula	PCI biometer	SS-OCT biometer	*P* value^*^
Tecnis ZCB00 (*n* = 102)			
SRK-T	− 0.128 ± 0.42	−0.136 ± 0.40	.59
Hoffer Q	−0.036 ± 0.46	−0.028 ± 0.44	.65
Holladay^b^	− 0.070 ± 0.44	−0.157 ± 0.34	.0003
Haigis	−0.046 ± 0.46	−0.038 ± 0.41	.69
AcrySof SA60AT (*n* = 48)			
SRK-T	0.008 ± 0.53	− 0.019 ± 0.52	.31
Hoffer Q	0.162 ± 0.51	0.126 ± 0.52	.28
Holladay^b^	0.127 ± 0.51	0.069 ± 0.46	.14
Haigis	0.128 ± 0.53	0.115 ± 0.47	.71

*PCI* partial coherence interferometry, *SS-OCT* swept-source optical coherence tomography

*Paired *t*-test

^a^Refractive mean error = postoperative spherical equivalent (SE) – predicted SE

^b^Holladay 1 and 2 formulas were embedded in PCI and SS-OCT biometers, respectively

**Table 3:** Comparison of refractive mean absolute error^a^ at 1 month after the surgery

	PCI biometer	SS-OCT biometer	*P* value^*^
Tecnis ZCB00 (*n* = 102)			
SRK-T	0.339 ± 0.28	0.334 ± 0.26	.69
Hoffer Q	0.364 ± 0.28	0.352 ± 0.26	.49
Holladay^b^	0.353 ± 0.27	0.334 ± 0.27	.40
Haigis	0.378 ± 0.26	0.335 ± 0.24	.03
AcrySof SA60AT (*n* = 48)			
SRK-T	0.399 ± 0.35	0.405 ± 0.31	.79
Hoffer Q	0.409 ± 0.33	0.401 ± 0.35	.77
Holladay^b^	0.410 ± 0.33	0.355 ± 0.30	.02
Haigis	0.417 ± .034	0.372 ± 0.31	.09

*PCI* partial coherence interferometry, *SS-OCT* swept-source optical coherence tomography

^*^Paired *t*-test

^a^Refractive mean absolute error = l postoperative spherical equivalent (SE) – predicted SE l

^b^Holladay 1 and 2 formulas were embedded in PCI and SS-OCT biometers, respectively

Table 4 shows the examination failure rate for both devices. The examination failure rate was higher in the PCI biometer than the SS-OCT biometer. The types of cataracts in eyes that could not be examined by at least one device are summarized in Table 5.

**Table 4:** Comparison of examination failure rate between the two devices

	Number of eyes			Failure rate (%)
	Failure	Success	Total	
PCI biometer	25	150	175	14.29
SS-OCT biometer	2	173	175	1.14

*PCI* partial coherence interferometry, *SS-OCT* swept-source optical coherence tomography

**Table 5:** Types of Cataract in eyes that failed to be examined by the devices

Type of cataract	Number of eyes	
	PCI biometer	SS-OCT biometer
Cortical opacity	1	0
ASC	2	0
PSC	18	0
ASC with PSC	2	0
Total white cataract	2	2
Total	25	2

*PCI* partial coherence interferometry, *SS-OCT* swept-source optical coherence tomography, *ASC* anterior subcapsular cataract, *PSC* posterior subcapsular cataract

## Discussion

This study showed significant differences in AL and Km measurements between SS-OCT and PCI biometers. Meanwhile, J0, J45 and ACD were not significantly different between the devices. The IOL power calculation in both devices was comparable when using SRK-T and Hoffer Q formula. The examination failure rate was lower when the SS-OCT rather than the PCI biometer was used.

A few studies have recently been published that evaluated a SS-OCT biometer compared with another one. Srivannaboon et al. [5] and Kathleen et al. [6] reported good agreement and excellent correlation between optical parameters measured by SS-OCT and PCI biometers. However, Akman et al. [7] and Yoo et al. [8] showed statistically significant differences in AL, K and ACD measurements between the SS-OCT and PCI biometers, although these differences were quite small for clinical significance. Our study supports these reports to some extent.

In this study, AL and ACD measured with the SS-OCT biometer were slightly shorter than those with the PCI biometer, with differences of 0.013 ± 0.03 mm and 0.023 ± 0.15 mm, respectively (***P*** < .0001, ***P*** = .06, respectively, paired *t*-test). The SS-OCT biometer measures the length of the optical pathway, such as AL, ACD, lens thickness and central corneal thickness, with SS-OCT technology [4]. In contrast, the PCI biometer measures AL and ACD using time-domain OCT technology and slit-imaging technology, respectively [4]. Light scattering by the retinal pigment epithelium and other ocular structures along with the visual axis is less than measured by conventional OCT, as SS-OCT uses a long wavelength of light. Therefore, the difference in AL measurements was likely caused by the difference in laser wavelength used in the two devices. Previous studies reported that 0.1 mm of AL difference would lead to a 0.28 D difference in the IOL power calculation and the minimum detectable change in refraction is 0.25 D with an AL of 0.075 mm [9, 10]. In that regard, even if there was a difference in AL measurements taken with the SS-OCT and PCI biometers in the present study, it was too small to cause a clinically significant error in IOL power calculation.

Measuring the K readings in both devices were based on the distance-independent telecentric keratometry system. SS-OCT measure K readings at 6 points each for 1.3, 2.4 and 3.2 mm diameter optical zones, while PCI-biometer uses only 6 points in a 2.4 mm zone [11, 12]. This would cause the differences in K readings between the devices in this study but those were quite small to make a clinical significance in IOL power calculation. However out data suggests that, in clinical practice, the differences in AL, K readings and ACD should be considered and those parameters could not be interchangeable between two devices. Meanwhile, the agreements of all the parameters (AL, Km, J0, J45_,_ and ACD) were generally good.

To assess the accuracy of the IOL power calculation, we compared ME and MAE at postoperative 1 week and 1 month when two different types of monofocal IOLs were used. The SRK-T, Hoffer Q, Holladay, and Haigis formulas are widely used for IOL power calculation. To predict the post-operative refractive error more accurately, the different formulas should be applied depending on the AL. For example, Hoffer Q is more accurate for shorter AL, while SRK-T is more suitable for long AL. However, out of total 47 eyes, only 2 eyes had short AL (< 22 mm) and 6 eyes had long AL (> 26 mm). Because the proportion of the eyes with extreme AL was too small, we didn’t divide the eyes depending on the AL. When using Holladay formula, the ME calculated with the SS-OCT biometer for Tecnis ZCB00 IOLs showed a tendency toward more myopia than those with the PCI biometer. This disparity in the tendency in ME between the two devices stemmed from the differences in the embedded Holladay formula in each device used for the present study. The other three formulas, for SRK-T, Hoffer Q, and Haigis, showed no statistically significant difference in either ME for Tecnis ZCB00 IOLs. In contrast, MAE showed a significant difference in Haigis formula for Tecnis ZCB00 IOLs (*p* = 0.0271, paired *t*-test). Unlike the 3rd generation formula, Haigis formula uses real ACD parameters to predict effective lens position. The tendency of ACD measurements to be shorter in SS-OCT biometer might cause the difference in MAE when using Haigis formula. In the other formulas, the MAE was not significantly different for Tecnis ZCB00 IOLs. In Acrysof SA60AT IOLs, ME and MAE were not significantly different in all formula between the two devices, except MAE calculated using Holladay formula. This disparity was also considered to be originated from the difference of embedded Holladay formula in each device.

In a few published studies, researchers described efforts to evaluate the accuracy of the IOL power calculation made with SS-OCT optical biometry [5, 13]. However, those studies did not use ME or MAE for the analysis. Srivannaboon et al. [5] compared predicted IOL power by the SRK-T and Haigis formulas and showed no significant differences between SS-OCT and PCI biometers. Arriola-Villalobos et al. [13] compared ocular parameters and calculated IOL power with the Holladay 2 and SRK-T formulas in SS-OCT and low-coherence reflectometry biometers. AL, ACD and Km were slightly different between the devices (mean differences = 0.0046 ± 0.022 mm, − 0.015 ± 0.038 mm, − 0.0546 ± 0.17 D, respectively; ***P*** = .09, .001, and .006, respectively). The calculated IOL power was also different when using the SRK-T formula, with a difference of 0.0517 ± 0.186 D (***P*** = .02).

A PCI biometer has been considered as a gold standard for IOL power calculation and widely used across the globe [14]. However, there are concerns over the limitations. One of the major concerns is the penetration ability that affected by the severity and type of cataracts. A PCI biometer uses dual-beam PCI with a 780 nm laser diode infrared light, whereas an SS-OCT biometer uses a 1055 nm tunable laser source [15]. The longer the wavelength a device uses, the less laser scatter it makes with better penetration ability. In this study, two eyes (1.14%) which showed total white cataract could not be examined with the SS-OCT biometer, whereas 25 eyes (14.29%) failed with the PCI biometer. Out of those 25 eyes, 20 had posterior subcapsular lens opacity and 4 had anterior subcapsular opacity. In short, SS-OCT exhibits much better penetration ability, especially in cases of ASC- or PSC-type cataracts. However, in white cataracts, both devices failed to measure AL.

To get a more accurate IOL power calculation, the A constant should be carefully considered. Basically, A constants used in optical biometers were adapted from the ULIB website [5]. However, to adjust the IOL power prediction, personalization by the surgeon based on large-scale clinical data analysis is important [16, 17]. In the present study, the A constant for the PCI biometer was previously optimized for MKK. However, the A constant used in the SD-OCT biometer was not personally optimized in the present analysis. After accumulating a large amount of clinical data on SD-OCT biometers, we can optimize the constant to improve the accuracy of IOL power calculation in clinical practice.

## Conclusions

In conclusion, the SS-OCT biometer seems to be comparable to the PCI biometer in measuring J0, J45 and ACD, whereas, AL and K readings were not comparable between two devices. Making IOL power calculation using SRK-T and Hoffer Q was comparable. Penetration ability is better with the SS-OCT biometer than the PCI biometer.

## Abbreviations

ACD: Anterior chamber depth
AL: Axial length
ASC: Anterior subcapsular cataract
IOL: Intraocular lens
K: Keratometry
Km: Mean keratometry
MAE: Mean absolute error
ME: Mean error
PCI: Partial coherence interferometry
PSC: Posterior subcapsular cataract
SS-OCT: Swept-source optical coherence tomography
